# New structural insight of C-terminal region of Syntenin-1, enhancing the molecular dimerization and inhibitory function related on Syndecan-4 signaling

**DOI:** 10.1038/srep36818

**Published:** 2016-11-10

**Authors:** Youngsil Choi, Ji-Hye Yun, Jiho Yoo, Inhwan Lee, Heeyoun Kim, Hye-Nam Son, In-San Kim, Ho Sup Yoon, Pascale Zimmermann, John R. Couchman, Hyun-Soo Cho, Eok-Soo Oh, Weontae Lee

**Affiliations:** 1 Department of Life Sciences, Division of Life and Pharmaceutical Sciences and the Research Center for Cellular Homeostasis, Ewha Womans University, Seoul 120-750, Korea; 2 Department of Biochemistry, College of Life Science & Biotechnology, Yonsei University, Seoul 120-749, Korea; 3 Department of Biology, College of Life Science & Biotechnology, Yonsei University, Seoul 136-791, Republic of Korea; 4 Biomedical Research Institute, Korea Institute of Science and Technology, Seoul 136-791, Republic of Korea; 5 Division of Structural and Computational Biology, School of Biological Sciences, Nanyang Technological University, Singapore; 6 Department of Genetic Engineering, College of Life Sciences, Kyung Hee University,Yongin-si Gyeonggi-do, 446-701, Republic of Korea; 7 Laboratory for Glycobiology, University of Leuven & Flanders Interuniversity Institute for Biotechnology, Leuven, Belgium; 8 Department of Biomedical Sciences, University of Copenhagen, Biocenter, 2200 Copenhagen, Denmark

## Abstract

The PDZ domain-containing scaffold protein, syntenin-1, binds to the transmembrane proteoglycan, syndecan-4, but the molecular mechanism/function of this interaction are unknown. Crystal structure analysis of syntenin-1/syndecan-4 cytoplasmic domains revealed that syntenin-1 forms a symmetrical pair of dimers anchored by a syndecan-4 dimer. The syndecan-4 cytoplasmic domain is a compact intertwined dimer with a symmetrical clamp shape and two antiparallel strands forming a cavity within the dimeric twist. The PDZ2 domain of syntenin-1 forms a direct antiparallel interaction with the syndecan-4 cytoplasmic domain, inhibiting the functions of syndecan-4 such as focal adhesion formation. Moreover, C-terminal region of syntenin-1 reveals an essential role for enhancing the molecular homodimerization. Mutation of key syntenin-1 residues involved in the syndecan-4 interaction or homodimer formation abolishes the inhibitory function of syntenin-1, as does deletion of the homodimerization-related syntenin-1 C-terminal domain. Syntenin-1, but not dimer-formation-incompetent mutants, rescued the syndecan-4-mediated inhibition of migration and pulmonary metastasis by B16F10 cells. Therefore, we conclude that syntenin-1 negatively regulates syndecan-4 function via oligomerization and/or syndecan-4 interaction, impacting cytoskeletal organization and cell migration.

Syndecans are type I transmembrane heparan sulfate proteoglycans found on most eukaryotic cell surfaces^1^. They comprise a large extracellular domain, a single transmembrane domain, and a short cytoplasmic domain. The extracellular domain is heavily glycosylated with heparan sulfate chains that functionally interact with numerous soluble and insoluble ligands in the pericellular environment^2^,^3^,^4^. The core protein sequences of the extracellular domains of various syndecans share little homology, providing functional diversity at the cell surface, whereas the transmembrane and cytoplasmic domains are highly conserved. The cytoplasmic tails have highly homologous regions proximal (C1) and distal (C2) to the membrane, with an intervening sequence (V region) that is specific and functionally relevant to each syndecan^5^. The presence of a divergent extracellular domain and a conserved cytoplasmic domain suggests that syndecans may have evolved to integrate diverse extracellular signals. We previously reported that both synthetic peptides and a recombinant cytoplasmic domain of syndecan-4 tend to oligomerize both *in vitro* and *in vivo*, and further showed that the solution structure of the non-phosphorylated cytoplasmic domain of syndecan-4 is a compact, intertwined dimer with a symmetrical clamp shape^6^,^7^. Since syndecans lack any intrinsic signaling capacity, the interactions of their cytoplasmic domains with various adaptor proteins can play essential regulatory roles. These adaptor proteins include the postsynaptic density protein, disc large, and the zonula occludens (PDZ) domain-containing scaffold protein, syntenin-1, which can link the cytoplasmic domain of certain syndecans to the cytoskeleton^8^.

Syntenin-1, a member of the PDZ domain-containing scaffold protein family, is involved in diverse physiological processes, such as cell adhesion^9^, protein trafficking^10^, and transcription factor activation^11^. The diverse biological functions of syntenin-1 arise from its ability to bind the cytoplasmic domains of numerous physiologically relevant signaling and adhesion molecules, including syndecans, neurexins, and ephrin B^12^,^13^,^14^,^15^. PDZ domains are found within diverse multidomain cytoplasmic proteins, where they play important roles in targeting the proteins to specific cell membrane receptors and channels, and assembling supramolecular signaling complexes^16^. Thus, syntenin-1 may contribute to regulating the function of syndecan by modulating the formation of its signaling complex.

Syntenin-1 is an adaptor-like molecule that contains at least four separate structural domains: An N-terminal domain, two tandem PDZ domains (PDZ1 and PDZ2), and a C-terminal region. A previous study showed that each syntenin-1 PDZ domain consists of two α-helices (α1 and α2) and six β-strands (β1-β6) that fold into a six-stranded β-sandwich^17^. X-ray crystallographic studies of syntenin-1 and a syndecan-4 cytoplasmic peptide suggested that the PDZ2 domain mainly recognizes the side chains of the FYA motif, while the C-terminus of the FYA-interacting peptide docks in a cavity formed by β2, α2, and a loop that exists between β1 and β2^17^. The syntenin-1 PDZ1 domain also binds to phosphatidylinositol 4,5-bisphosphate (PIP2), in an interaction that controls the association of syntenin-1 with the plasma membrane^18^. The high-affinity interaction between syntenin-1 and syndecans-4 requires the tandem PDZ domains of syntenin-1^14^. The membrane-syntenin-1 complex with the highest stability consists of the PDZ1 and PDZ2 domains engaging PIP2 and syndecans-4, respectively^14^,^18^. These findings suggest that a syndecan-4/syntenin-1 complex forms in the proximal membrane region. However, we do not yet fully understand the regulatory mechanism of this interaction.

We previously showed that the syndecan-4 cytoplasmic domain interacts with PIP2 and protein kinase Cα (PKCα) through the V region to regulate downstream functions of syndecan-4, such as the formations of focal adhesions and actin stress fibers^19^,^20^,^21^. Collectively, these previous studies suggested that syntenin-1-mediated scaffolding might play a critical role in the syndecan-4-mediated regulation of cytoskeletal organization. Here, we provide novel evidence that syntenin-1 interacts with syndecan-4, thereby inhibiting the binding between PKCα and syndecan-4 and negatively regulating the functions of syndecan-4

## Results

### Two syntenin-1 dimers form a complex with a dimer of syndecan-4

**Figure 1:**
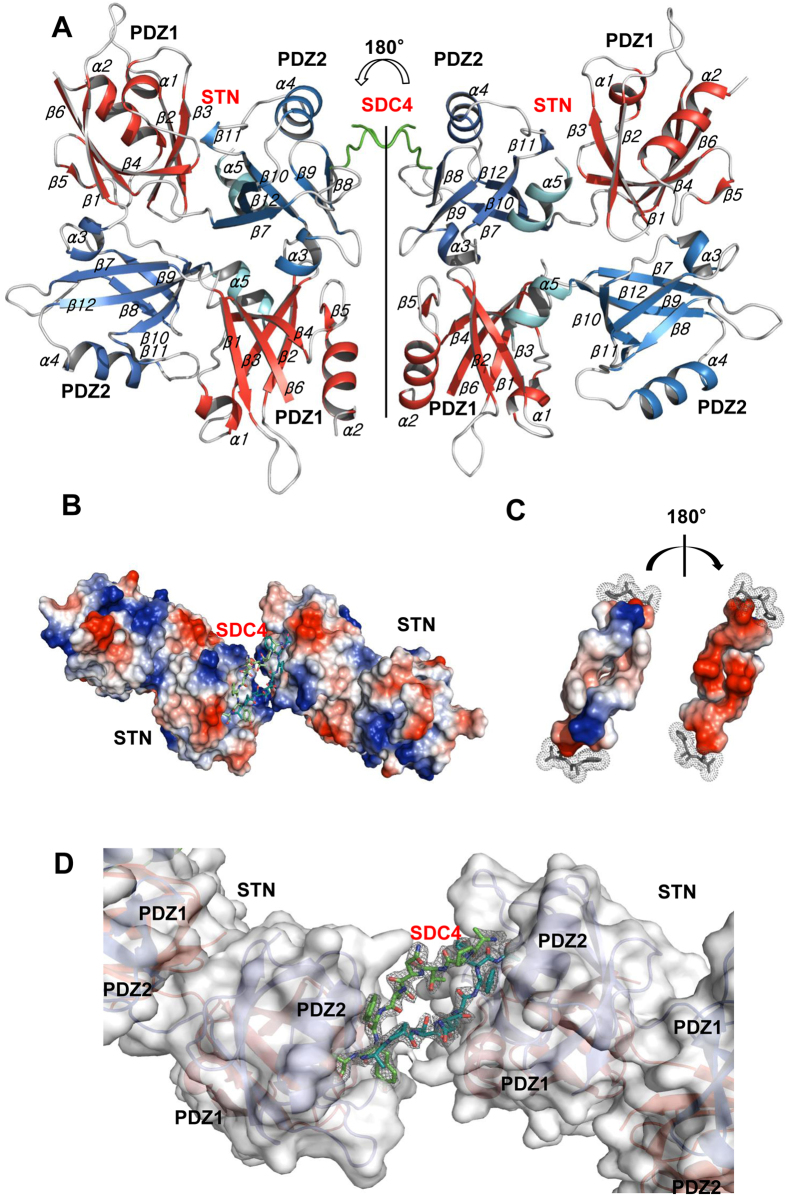
Crystal structure of the syntenin-1/syndecan-4 complex. (**A**) The crystal structure of the syntenin-1/4C2 complex is shown as a ribbon diagram. PDZ 1 and PDZ 2 domains are displayed in red and blue, respectively. One C-terminal extra helix is colored by cyan. Syndecan-4 dimer is drawn in green lines. (**B**) Surface charge model of syntenin-1 dimer with a bridge formed by syndecan-4 is demonstrated. (**C)** Surface charge of syndecan-4 shows opposite charge distribution of dimeric surfaces. **(D)** The electron density map of the syndecan-4 dimer in syntenin-1/4C2 complex. Syndecan-4 (SDC4) cytoplasmic domain wraps the syntenin-1 dimers. The syndecan-4 cytoplasmic domain forms an antiparallel dimer with a twist in the terminal region. The map is contoured at 2.0σ with a cover radius of 2.2 Å applied.

Although the primary sequence and secondary structures of syntenin-1 are well conserved across vertebrates^9^, no previous study had reported detailed structure-function relationships for this protein. Here, we first investigated the intermolecular interactions between syntenin-1 and syndecan-4. The crystal structures of syntenin-1 with and without a syndecan-4 cytoplasmic domain peptide (corresponding to the C2 region; 4C2) were determined to be 2.8 and 1.9 Å, respectively (Table 1). Surprisingly, our analysis of the syntenin-1/4C2 complex revealed that syntenin-1 formed a symmetrical pair of dimers, each of which was anchored by a syndecan-4 dimer (Fig. 1A). Each syndecan-4 bridged two syntenin-1 dimers, forming a unique dimer-of-dimers topology (Fig. 1B). In terms of its overall conformation, the syndecan-4 cytoplasmic domain exists as a compact intertwined dimer with a symmetrical clamp shape, and its molecular surface is highly charged (Fig. 1C). The two parallel strands of the syndecan cytoplasmic domain form a cavity in the center of the dimeric twist (Fig. 1D). In our structural superpositioning of syntenin-1/4C2 with syntenin-1, the overall RMSD was 1.493 Å. In the absence of syndecan-4 however, the molecular surface of PDZ2 in the syntenin-1/4C2 complex was wider than that of syntenin-1. Upon syndecan-4 binding, a structural rearrangement of the PDZ2 domain provided a specific binding pocket for the syndecan-4 cytoplasmic domain (Fig. S1a and S1b in supplementary information). PDZ1 helix-2 (α2) also underwent rearrangement upon syndecan-4 binding (Fig. S1a and S1c in supplementary information). Thus, data from X-ray crystallography and nuclear magnetic resonance (NMR) spectroscopy of the syntenin-1/syndecan-4 complex suggest that a symmetrical syntenin-1 dimer-of-dimers is anchored near the membrane by a syndecan-4 dimer. These findings may provide insight into the relative arrangement of the syntenin-1/syndecan-4 complex *in vivo*.

### Syntenin-1 negatively regulates the functions of syndecan-4

The interaction between PKCα and the central V region of syndecan-4 is a critical step in actin cytoskeleton rearrangement and focal adhesion formation. Because the binding sites for PKCα and syntenin-1 are positioned very close together in the syndecan-4 cytoplasmic domain, we investigated whether the binding of syntenin-1 to syndecan-4 affects that of PKCα. Rat embryonic fibroblasts (REFs) were co-transfected with syndecan-4 and either empty vector or a vector encoding the HA-tagged syntenin-1 cDNA, and immunoprecipitates were immunoblotted with an anti-PKCα antibody. Our results revealed that syntenin-1 expression competitively inhibited the interaction of PKCα with syndecan-4 (Fig. S2a in supplementary information). To further examine the effect of syntenin-1 on the association between syndecan-4 and the PKC catalytic domain (PKM) at the cellular level, we performed a fluorescence resonance energy transfer (FRET) assay using REFs expressing fluorescent protein-fused syndecan-4 and PKM. FRET between syndecan-4-YFP and PKM-CFP was detected after acceptor (YFP) photobleaching (6.39 ± 0.53%), suggesting that syndecan-4 interacts with PKM *in vivo* (Fig. S2b and S2c in supplementary information). Consistent with the above described *in vitro* data, the efficiency of FRET between syndecan-4 and PKM was significantly reduced by syntenin-1 overexpression (−3.24 ± 0.53%, Fig. S2c in supplementary information). In addition, syntenin-1 expression reduced the syndecan-4-mediated phosphorylation of PKCα in REFs (Fig. S2d in supplementary information). These data suggest that syntenin-1 may contribute to the regulation of syndecan-4-mediated focal adhesion-related signaling. In addition, REF cells were co-transfected with vectors encoding either syndecan-4 alone or syndecan-4 plus syntenin-1, and focal adhesions were detected by assessment of paxillin distribution. Consistent with a previous report^22^, increased focal adhesion formation was observed in syndecan-4-transfected REF cells compared with vector-transfected cells (Fig. S2e in supplementary information). In contrast, there was no increase in focal adhesion formation among REF cells co-expressing syntenin-1 and syndecan-4 (Fig. S2e in supplementary information), suggesting that syntenin-1 inhibits the syndecan-4-mediated focal adhesion signaling pathway. Moreover, syndecan-4 negatively regulated the migration of REFs, whereas co-expression of syntenin-1 overcame this effect (Fig. S2f in supplementary information). Together, these data suggest that syntenin-1 plays an important role in negatively regulating the signaling functions of syndecan-4.

### A molecular interaction between the PDZ2 domain of syntenin-1 and syndecan-4 is important for the inhibition of syndecan-4

To further investigate the interaction between syntenin-1 and the cytoplasmic domain of syndecan-4 in solution, we performed surface plasmon resonance (SPR) and NMR spectroscopy experiments. A dissociation constant of 509 nM was determined for the binding of syntenin-1 to the syndecan-4 cytoplasmic domain (Fig. S3a in supplementary information). Our NMR data suggested that the syndecan-4 cytoplasmic domain bound strongly to PDZ2, which is consistent with a previous report^18^. The PDZ2 domain triggered a much greater chemical shift perturbation than that of PDZ1 in solution (Fig. S3b and S3c in supplementary information), suggesting that syndecan-4 mainly interacts with the syntenin-1 PDZ2 domain under physiological conditions. Consistent with this notion, PDZ2 (but not PDZ1) reduced the interaction of syndecan-4 with PKCα (Fig. S4a in supplementary information). Furthermore, expression of the PDZ2 domain significantly reduced the membrane localization (Fig. S4b in supplementary information) and activity (Fig. S4c in supplementary information) of PKCα. Finally, the PDZ2 domain rescued cell migration more efficiently than the PDZ1 domain (Fig. S4d in supplementary information). Together, these data provide strong evidence that the PDZ2 domain is essential for the regulatory functions of syntenin-1.

### A direct interaction is crucial for the ability of syntenin-1 to attenuate syndecan-4 signaling

**Figure 2:**
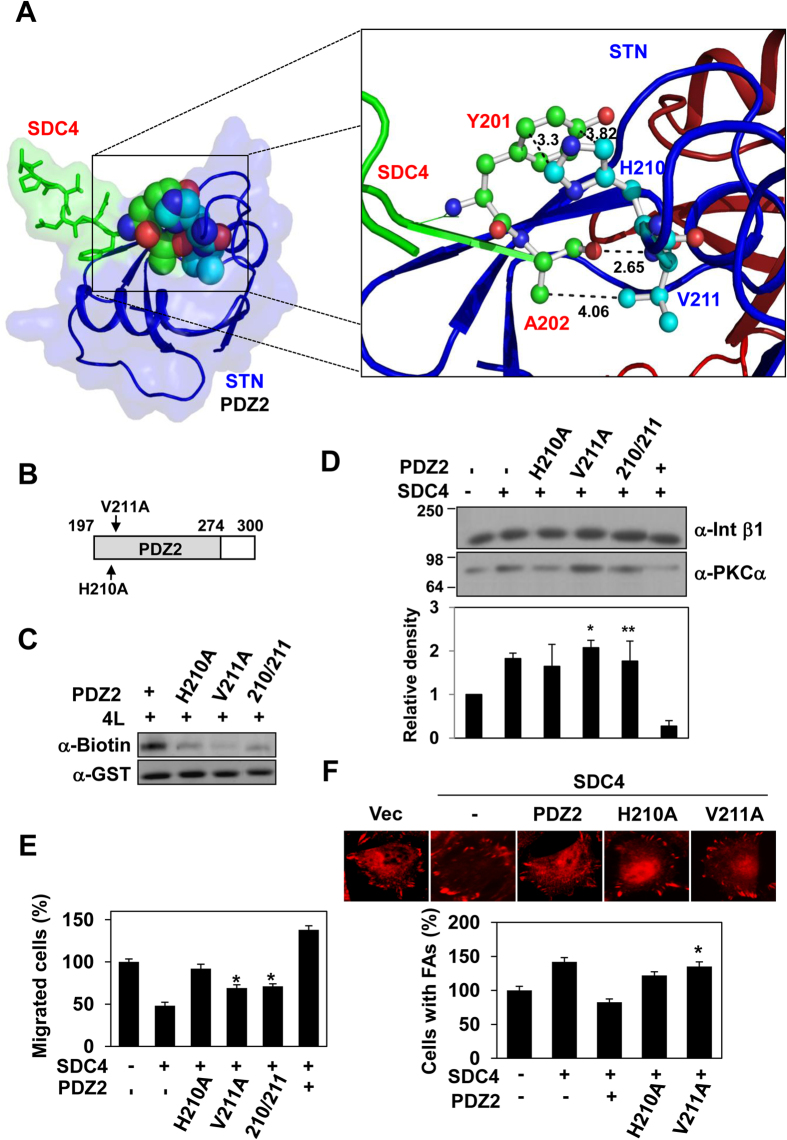
Syntenin-1 as a negative regulator of syndecan-4 via the PDZ2 domain. (**A**) Intermolecular interactions between syntenin-1 (STN) and syndecan-4 (SDC4) are presented. Hydrophobic residues involved in the interaction were identified and displayed using the Pymol program. Syndecan-4 Y201 and A202 residues (red) interact closely with syntenin-1 residues H210 and V211 (blue), indicating close side chain distance in the range of 2.65 to 4.06 Å. (**B**) Schematic representation of PDZ2 mutants. The key residues in the PDZ2 domain were substituted with Ala (arrows). (**C**) Purified GST-PDZ2 or -PDZ2 mutant proteins were incubated with biotinylated syndecan-4 cytoplasmic domain peptides (4 L) for 2 hr. Bound proteins were collected and detected by immunoblotting with anti-biotin antibody. (**D**) REF cells were co-transfected with the indicated cDNAs and the amount of PKCα in the membrane fraction was determined by immunoblotting with anti-PKCα antibody. Protein levels were quantified by densitometric scanning (bottom panel). The mean of protein expression ± SEM is shown; *P < 0.01. **p < 0.05 (**E**) Transwell migration assays were performed using FGF-2 (10 ng/ml) as a chemoattractant in the lower chamber. Transfected cells were allowed to migrate on gelatin-coated (10 μg/ml) Transwell plates for 24 hr. Representative results from three independent experiments are shown. *p < 0.01 vs. PDZ2. (**F**) REF cells were co-transfected with SDC4 and either PDZ2 or its mutants. After 24 hr, cells were fixed with 3.5% PFA for 5 min, extracted with 0.1% Triton X-100 in PBS for 10 min, and stained for the focal adhesion component paxillin *(top panel)*. Percentages of cells showing focal adhesions (mean ± SE; vector transfectants set as 100%, *lower panel*). *p < 0.01 vs. PDZ2.

Our crystal structure analysis of the syntenin-1/4C2 complex showed that a major interaction between syntenin-1 and syndecan-4 originated from hydrophobic forces between syndecan-4 residues, Y201 and A202, and syntenin-1 residues, H210 and V211 (Fig. 2A). When these syntenin-1 residues were mutated to alanines (Fig. 2B), the interaction of syntenin-1 with a synthetic peptide corresponding to the entire cytoplasmic domain of syndecan-4 (4L) was notably reduced (Fig. 2C). A more marked reduction occurred with the V211A mutant than the H210A mutant, implying that V211 is more critical for the intermolecular interaction with syndecan-4. Consistent with this finding, whereas syntenin-1 significantly reduces membrane localization of PKCα, the V211A mutant does not show this effect (Fig. 2D). The V211A mutant could not overcome the syndecan-4-mediated reduction in cell migration, whereas the H210A mutant yielded a recovery of cell migration (Fig. 2E). Similarly, focal adhesion formation in V211A-expressing REFs showed less of a decrease than that seen in PDZ2-expressing REFs (Fig. 2F). Together, these data strongly suggest that syntenin-1 negatively regulates syndecan-4 signaling via a direct interaction with syndecan-4.

### Syntenin-1 dimerization is crucial for the negative regulation of syndecan-4

**Figure 3:**
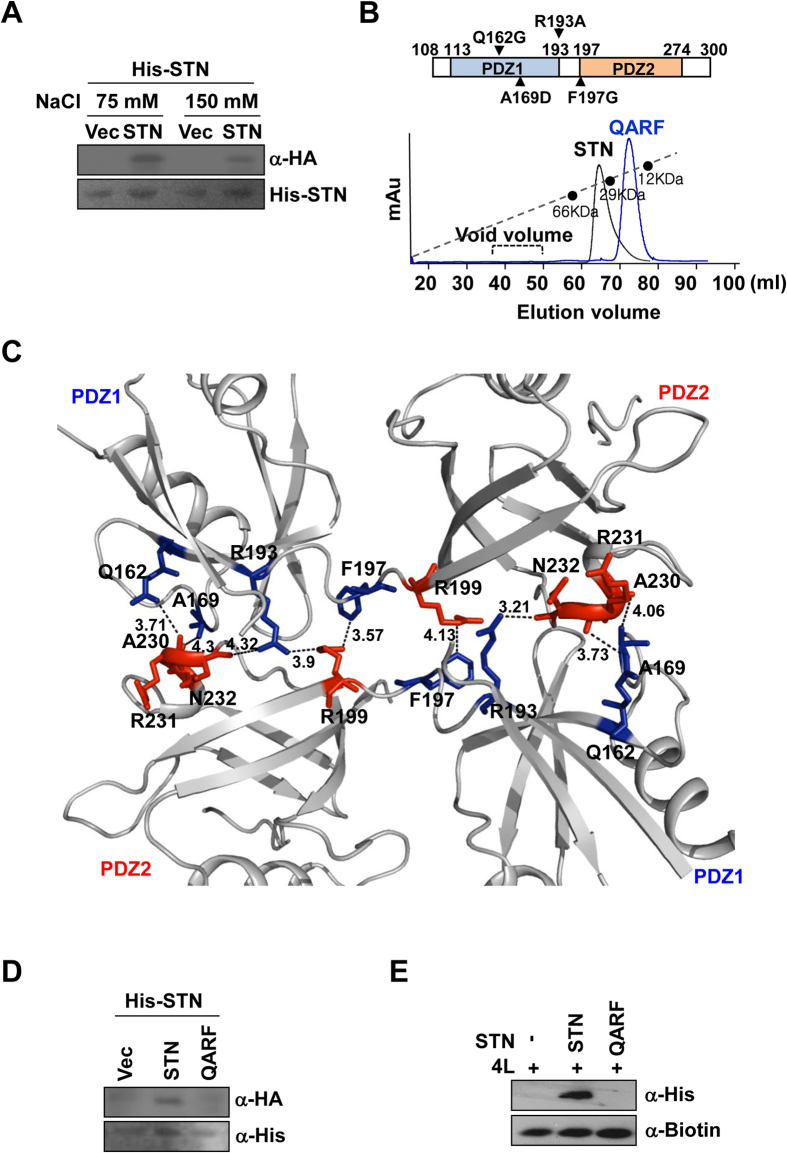
Dimer interface and molecular interactions of syntenin-1. (**A**) Purified His-tagged syntenin-1 proteins (His-STN) were incubated for 2 hr with lysates of REF cells transfected with empty vector (Vec) or HA-tagged STN. Bound proteins were collected and immunoblotted with anti-HA antibody. (**B**) Schematic representation of syntenin-1 mutants (*top panel*). Gel filtration chromatography was performed using HiLoad 16/600 Superdex 75 prep grade column calibrated by blue dextran (2000 KDa), albumin (66 KDa), carbonic anhydrase (29 KDa) and cytochrome C (12.4 KDa) (*bottom panel*). The elution of protein standards is shown. The molecular weight (Mw) is calculated using the equation, log*y* = −1.2177*x* + 6.263, where *x* represents V*e* (experimental elution volume)/V*o* (void volume; 45.85 ml). Analyzed molecular weight was described in Table 2. (**C**) Dimerization interface of syntenin-1 shows that both hydrophobic and electrostatic interactions between PDZ1 and PDZ2 domains play important roles in dimer formation. (**D**) Purified His-tagged STN proteins were incubated for 2 hr with lysates of REF cells transfected with Vec, HA-tagged STN, or dimerization-defective mutant (QARF). Bound proteins were collected and immunoblotted with anti-HA antibody. (**E**) Equal amounts of biotinylated-4 L were incubated with His-tagged STN or -QARF proteins for 2 hr. Proteins bound were immunoblotted with anti-His antibodies.

**Figure 4:**
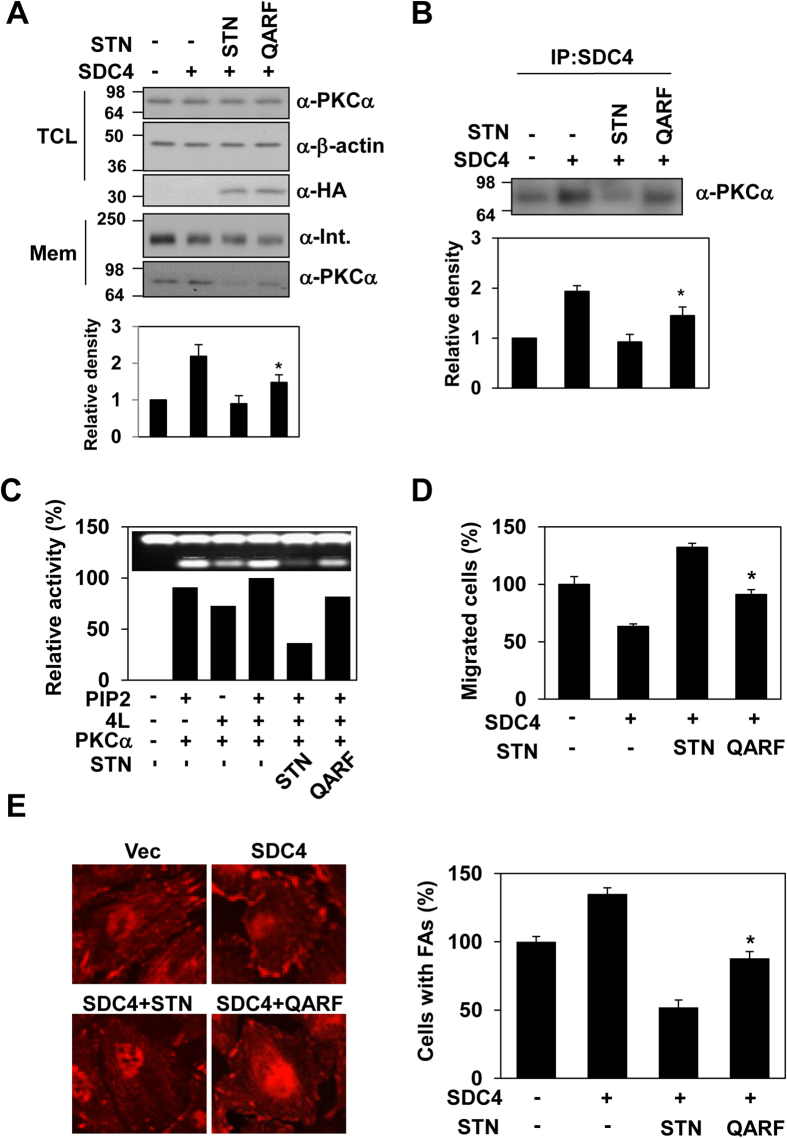
Dimeric syntenin-1 in the regulation of syndecan-4 functions. (**A**) Total cell lysates (TCL) were separated by electrophoresis on 12% SDS-PAGE gels and syntenin expression was analyzed by immunoblotting with anti-HA antibody. Cells were fractionated, and proteins in the membrane fraction (Mem) were resolved by SDS-PAGE and subjected to immunoblotting with antibodies against PKCα and α-integrin β1 (α-Int.). PKCα in nuclear fraction was quantified by densitometric scanning (bottom panel). The mean of protein expression ± SEM is shown; *P < 0.01. (**B**) Transfected cells were immunoprecipitated with anti-syndecan-4 antibody and immune complexes were immunoblotted with anti-PKCα. PKCα protein levels were quantified by densitometric scanning (bottom panel). The mean of protein expression ± SEM is shown; *P < 0.01. (**C**) PKC assays were performed as described in Online Methods with purified His-STN or QARF. Relative activity is indicated by mean ± SE (n = 5) compared with that in the presence of syndecan-4 proteins with PIP2. (**D**) Cell migration assays were performed as described in Fig. 3E. Representative results of three independent experiments are shown. *p < 0.01 vs. syntenin-1. (**E**) REFs co-transfected with the indicated cDNAs were fixed, permeabilized, and stained with anti-paxillin antibody, as described in Fig. 3F *(left panel)*. Percentages of cells showing focal adhesions (mean ± SE, *right panel*). *p < 0.01 vs. syntenin-1.

The crystal structure of the syntenin-1/4C2 complex suggests that dimerization might be important for the function of syntenin-1. Indeed, both pull-down assays (Fig. 3A and Table 2) and gel filtration chromatography (Fig. 3B) showed that syntenin-1 exists as a dimer under physiological conditions. Our structural analysis showed that four residues (Q162 and A169 in PDZ1; R193 and F197 in the linker region) were involved in dimeric interactions with four PDZ2 residues (R199, N232, A230, and R231; Fig. 3C). Notably, both electrostatic and hydrophobic interactions contribute to the dimerization of syntenin-1. Based on this structural information, we constructed the dimerization-incompetent quadruple mutant of syntenin-1, QARF (Q162G, A169D, R193A, and F197G). As expected, QARF failed to dimerize (Fig. 3B,D and Table 2) and exhibited a reduced interaction with syndecan-4 (Fig. 3E). QARF also failed to inhibit the functions of syndecan-4, such as the syndecan-4-mediated membrane localization of PKCα (Fig. 4A), the interaction of syndecan-4 with PKCα (Fig. 4B), and the syndecan-4-mediated activation of PKCα (Fig. 4C). In addition, QARF mutants were much less efficient than wild-type syntenin-1 in inhibiting syndecan-4-mediated cell migration (Fig. 4D), and were found to increase focal adhesion formation (Fig. 4E). These results support the hypothesis that the dimerization of syntenin-1 is essential for its ability to regulate the functions of syndecan-4.

**Figure 5:**
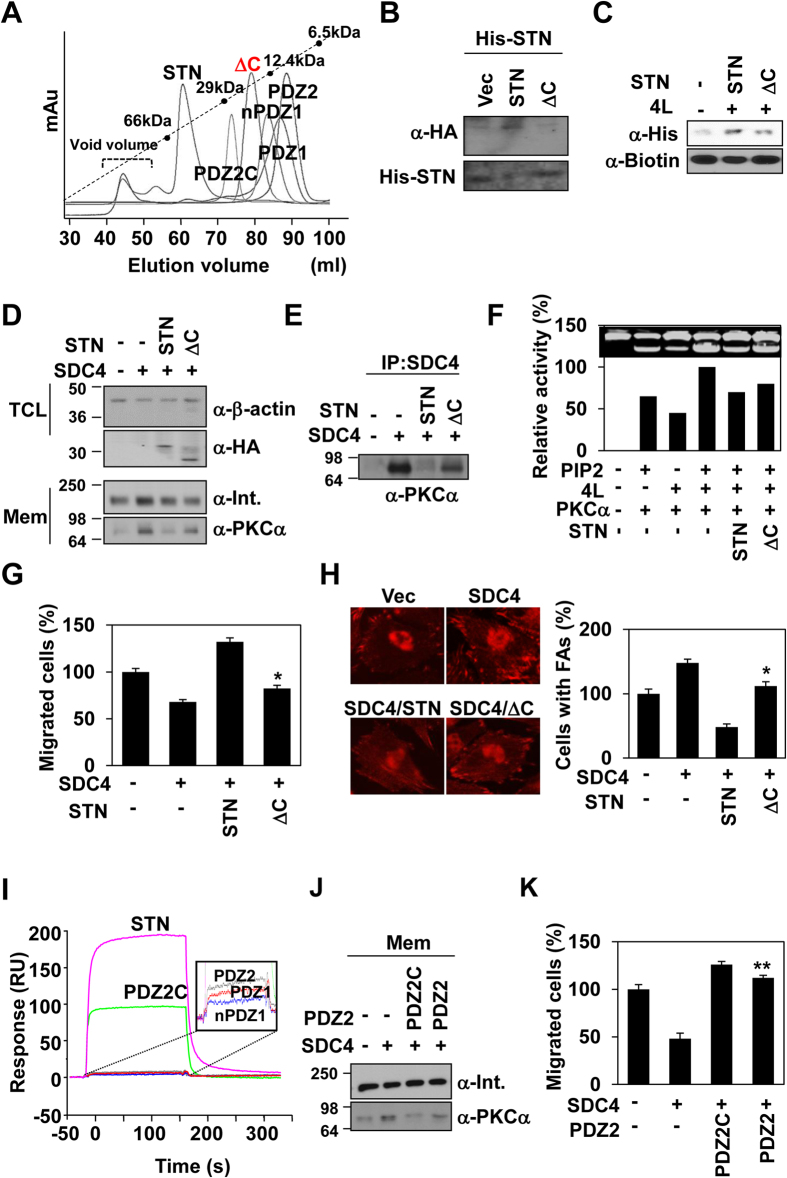
The role of C-terminal domain of syntenin-1 for dimerization and negative regulation of syndecan-4. (**A**) Gel filtration chromatography was performed using HiLoad 16/600 Superdex 75 prep grade column calibrated by albumin (66 KDa), carbonic anhydrase (29 KDa), cytochrome C (12.4 KDa) and aprotinin (6.5 KDa). The elution of protein standards is shown. The molecular weight (Mw) is calculated using the equation, log*y* = −0.0267*x* + 3.2833, where *x* represents the experimental elution volume. Analyzed molecular weight was described in Table 3. (**B**) Pull-down assay was performed as described in Fig. 4A. (**C**) Pull-down assay was performed as described in Fig. 3E. (**D**) Cells were co-transfected with the indicated cDNAs and total cell lysates (TCL) or membrane preparations (Mem) were analyzed by immunoblotting with antibodies shown. (**E**) Immunoprecipitation was performed as described in Fig. 5B. (**F**) PKC assays were performed as described in Fig. 5C. Relative activity is indicated by mean ± SE (n = 5) compared with that in the presence of syndecan-4 with PIP2. (**G**) Migration assays were performed as described in Fig. 3E. Data represent the average of at least three independent experiments; *p < 0.01 vs. syntenin-1. (**H**) Transfected cells were stained with anti-α-paxillin antibody as described in Fig. 3F *(left panel)*. Percentages of cells containing focal adhesions (mean ± SE; vector transfectants set as 100%, *right panel*). Results are representative of three separate experiments; *p < 0.01 vs. syntenin-1. (**I**) Profiles of 4 L binding to STN or deletion mutants immobilized on a carboxymethyl dextran surface. Signals are presented as a plot of resonance units (RUs) vs. time. (**J**) Cells were transfected with the indicated cDNAs and the amount of PKCα in the membrane fraction was determined by immunoblotting with indicated antibodies. (**K**) Migration assays were performed as described in Fig. 3E. Data are representative of five independent experiments. **p < 0.05 vs. PDZ2C.

Gel filtration column chromatography showed that constructs containing the syntenin-1 C-terminal region (STN-1 and PDZ2C) but not those lacking the syntenin-1 C-terminal region (PDZ1, PDZ2, nPDZ1 and STNΔC) were able to undergo dimerization (Fig. 5A and Table 3), suggesting that the C-terminal region of syntenin-1 mediates its dimerization. Consistent with this notion, deletion of the C-terminal region (STNΔC) markedly reduced the intermolecular interactions of syntenin-1 with syntenin-1 (Fig. 5B) and syntenin-1 with syndecan-4 (Fig. 5C). Furthermore, C-terminal truncated syntenin-1 (STNΔC) mutant could not efficiently block various syndecan-4-mediated effects, including PKCα membrane localization (Fig. 5D), the interaction with/activation of PKCα (Fig. 5E,F), the inhibition of cell migration (Fig. 5G), and focal adhesion formation (Fig. 5H). These data strongly suggest that the syntenin-1 C-terminal region is essential for the dimerization and functional abilities of syntenin-1. A PDZ2 construct containing the C-terminal tail (PDZ2C) interacted with syndecan-4 with higher affinity than the truncated form (PDZ2; Fig. 5I). Deletion of the C-terminal region rendered PDZ2 less efficient at reducing the membrane localization of PKCα (Fig. 5J) and enhancing cell migration (Fig. 5K).

**Figure 6:**
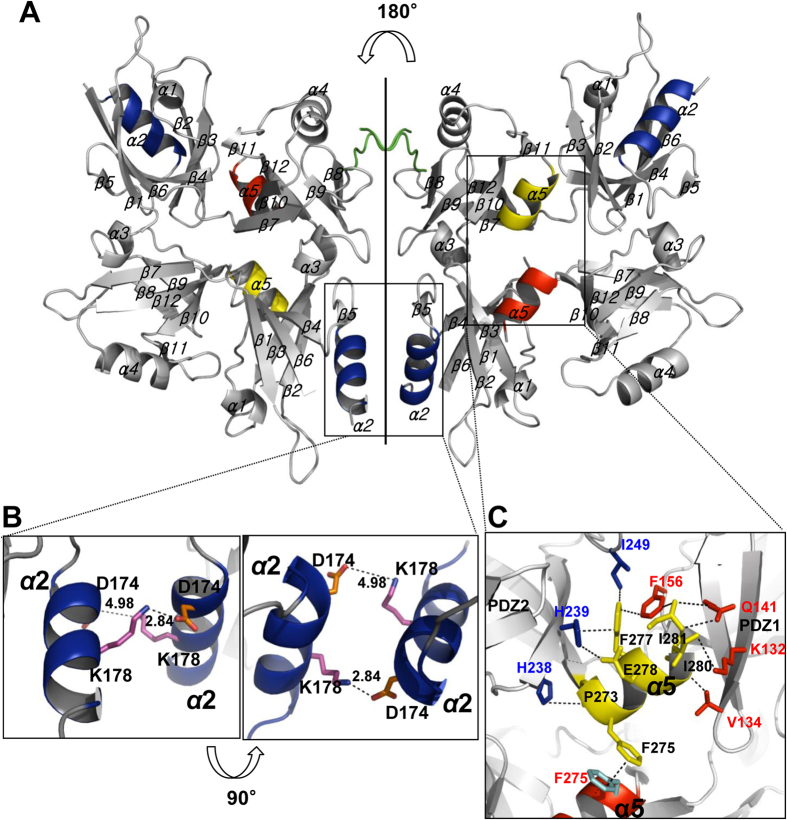
C-terminal helix of syntenin-1 induces compact dimeric and tetrameric interaction of the syntenin-1 with syndecan-4 complex. (**A**) Crystal structure is shown as a ribbon diagram. Monomeric syntenin-1 has five α-helices and twelve β-strands. The syntenin-1/4C2 complex comprises the 4C2 dimer and 4C2 mediated syntenin-1 tetramer. The flexible residues in the C-terminal region were not observed in the electron density map. (**B**) Expanded region of the syntenin-1/4C2 complex. Two salt bridges are present between D174 and K178 of apposed α2 helices of PDZ1. (**C**) Helix 5 of syntenin-1 interacts with both the PDZ1 and the PDZ2 domain. Residues P273, F277, and E278 of helix 5 are involved in side chain interactions with H238, H239, and I249 of the PDZ1 domain. Residues I280 and I281 interact directly with residues V134, K132, Q141, and F156 of the PDZ2 domain.

Structurally, the C-terminal region of syntenin-1 was found to form a short α-helix (α5), and residues P273, F277, E278, I280, and I281 of α5 were observed to participate in molecular interactions with residues of PDZ1 and PDZ2, thereby stabilizing the syntenin-1/4C2 complex. Notably, the F275 of one α5 interacted with that of the other subunit (Fig. 6A,C). Moreover, the C-terminal region of syntenin-1 induced a large conformational change that triggered inter-dimeric interactions between the α2 helices of the PDZ1 domains with a salt bridge between D174 and K178 (Fig. 6A,B). We further determined the crystal structure of C-terminal truncated syntenin-1 (STNΔC) in the presence of the syndecan-4 cytoplasmic domain peptide (4C2) at a resolution of 2.5 Å (Table 1), and found that the overall folds were very similar between syntenin-1/4C2 and STNΔC/4C2. Although the molecular topologies of PDZ1 and PDZ2 were very similar, a number of structural differences were observed: the helix-2 of the PDZ1 domain (α2) was perturbed due to an antiparallel interaction between D174 and K178; and the short α-helix (α5) interacted with PDZ1 and α5 of the other subunit (Fig. 6B,C). The latter interaction contributed to stabilization of the syntenin-1/4C2 complex. Therefore, the C-terminal region of syntenin-1 seems to contribute to the homodimerization and syndecan-4 mediated oligomerization of syntenin-1.

### Syntenin-1 negatively regulates the tumor suppressor role of syndecan-4 in melanoma

**Figure 7:**
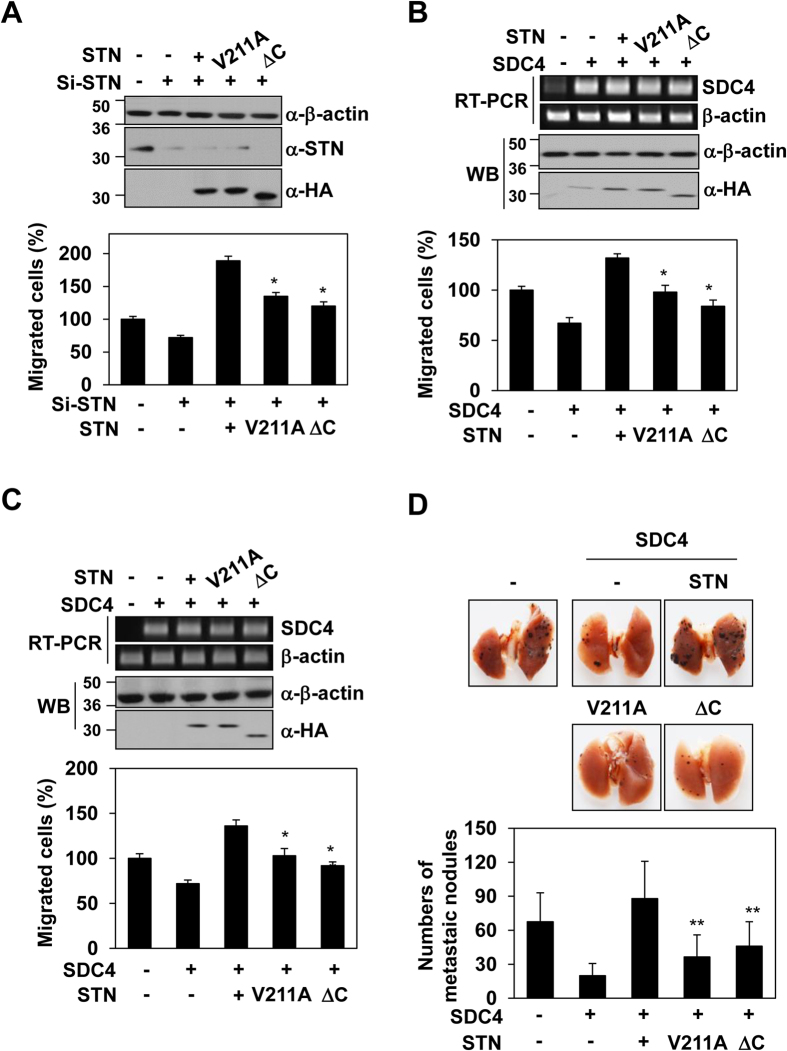
The role of the syndecan-4/syntenin-1 complex in the tumorigenic regulation of melanoma cells. (**A**) A375 melanoma cells were transfected with the indicated cDNAs, and the expression levels of syntenin-1 proteins were evaluated by Western blotting (*top panel*). Transfected cells were allowed to migrate on gelatin-coated (10 μg/ml) Transwell plates (*bottom panel*). (**B**,**C**) A375 melanoma cells (**B**) or human epidermal melanocytes (HEMs) were transfected with the indicated cDNAs, and expression level of syndecan-4 mRNA and syntenin-1 proteins were evaluated by RT-PCR and western blotting, respectively (*top panel*). Transfected cells were allowed to migrate on gelatin-coated (10 μg/ml) Transwell plates (*bottom panel*). *p < 0.01 vs. syntenin-1. (**D**) C57BL/6 mice were intravenously (i.v.) injected with B16F10 cells (1 × 10^5^ cells) stably expressing the indicated cDNAs. Thirteen days post-injection, the surface metastatic nodules in the lungs were photographed (*top panel*) and counted (*bottom panel*). **p < 0.05 versus syntenin-1.

Syntenin-1 is overexpressed in multiple human cancer cell lines, including melanoma cell lines, and has been associated with increased cell migration and invasion^23^. Syndecan-4 is also known to mediate the invasion and metastasis of melanoma cells^24^. However, no previous study has reported a comprehensive analysis of the potential crosstalk between syntenin-1 and syndecan-4 in melanoma. To first assess the role of syndecan-4 in melanoma cells, we examined the effects of syndecan-4 expression on melanoma cell migration. Overexpression of syndecan-4 significantly reduced the migration of A375 melanoma cells (Fig. S5a in supplementary information), whereas siRNA-mediated reduction of syndecan-4 expression enhanced the migration of these cells (Fig. S5b in supplementary information). Consistent with this finding, overexpression of syndecan-4 reduced the pulmonary metastatic potential (Fig. S5c in supplementary information) and popliteal lymph node metastasis (Fig. S5d in supplementary information) of B16F10 melanoma cells in mice. These findings strongly suggest that syndecan-4 inhibits the cancer-associated cell migration of melanoma cells. Furthermore, overexpression of syntenin-1 also enhanced the migration of B16F10 cells (Fig. 7A), whereas siRNA-mediated knockdown of syntenin-1 significantly reduced the migration of these cells (Fig. 7B). This confirms that syntenin-1 positively regulates melanoma cell migration.

To investigate the potential crosstalk between syntenin-1 and syndecan-4, we next compared their expression levels with the extent of cell migration. A375 human melanoma cells were subjected to siRNA-mediated knockdown of human syntenin-1 and transfected with vectors encoding wild-type mouse syntenin-1 (STN), an interaction-defective mutant (V211) syntenin-1 or a dimerization-defective mutant (ΔC) syntenin-1. Similar to our above-described results, the V211 and ΔC mutants failed to inhibit the syndecan-4-mediated migration of A375 melanoma cells (Fig. 7A). Consistent with this, the overexpression of syntenin-1 but not its mutants rescued the syndecan-4-mediated reduction in the migration of A375 melanoma cells (Fig. 7B) and human melanocytes (Fig. 7C), suggesting that the syndecan-4/syntenin-1 interaction is important for melanoma cell migration. Indeed, analysis of lung samples revealed that mice injected with syntenin-1 mutant-transfected B16F10 cells developed significantly fewer pulmonary metastases than those injected with wild-type syntenin-1-transfected cells (Fig. 7D). Together, these data indicate that syntenin-1 negatively regulates syndecan-4-mediated anti-tumorigenic activity in melanoma cells.

## Discussion

Although syntenin-1 is a known syndecan-binding protein^8^, apart from knowing that this interaction has potential roles in trafficking^25^ and exosome formation^26^, we do not yet fully understand the relevant molecular functions. The present study provides clear evidence that syntenin-1 negatively regulates syndecan-4 signaling via binding between syndecan-4 and the syntenin-1 PDZ2 domain. In cells, our data demonstrate that syntenin-1 reduced the association of syndecan-4 with PKCα and decreased the ability of syndecan-4 to activate PKCα. Therefore, syntenin-1 overexpression blocked the functions of PKCα is primary fibroblast focal adhesions, reduced the level of syndecan-4-mediated focal adhesion formation, and rescued the syndecan-4-mediated inhibition of cell migration.

This interplay between syndecan-4 and syntenin-1 may provide a means through which the functions of syndecan-4 are regulated during cell adhesion. Indeed, syntenin-1 blocked most functions of syndecan-4, including the enhancement of focal adhesion formation and the reduction of cell migration. At a molecular level, this inhibition may be elicited at several levels. First, it might be an effect of steric hindrance. Although the binding site for syntenin-1 does not directly overlap that of PKCα, an array of four syntenin-1 molecules might fill the space around the variable region (Fig. 1). Second, since the molecular topology of syntenin-1/4C2 is planar with respect to the membrane and the PDZ1 domain interacts with PIP2, it may compete with PKCα for this inositide. Third, the interaction of syntenin-1 may block the formation of higher-order oligomers of syndecan-4. The crystal structure of the syntenin-1/4C2 complex revealed a bona fide architecture in which the syndecan-4 dimer bridges the dimer-of-dimers of syntenin-1 (Fig. 1A). Since the oligomeric status of the syndecan-4 cytoplasmic domain is critical for the interaction of syndecan-4 with PKCα^27^, the reduction in PKCα activity triggered by the binding of syntenin-1 to syndecan-4 inhibits various syndecan-4-mediated functions, including focal adhesion formation.

Structural analysis of the individual PDZ domains of syntenin-1 suggested that Phe and Tyr residues in the syndecan-4 C2 region play critical roles in the interaction with the PDZ2 domain of syntenin-1. The Tyr residue of the FYA peptide is cushioned by His-208, Ile-212, and Val-222. Notably, the aromatic ring of the tyrosine residue is also involved in an off-center stacking interaction with the critical His-208 residue of the PDZ2 domain.

Previous experiments have shown that the highly invasive/metastatic cancer cell lines, MDA-MB-435 and MDA-MB-235, express more syntenin-1 than the poorly invasive/metastatic cancer cell lines, MCF-7, MDA-MB-453, MDA-MB-468, and T47D^28^. Moreover, exogenous expression of syntenin-1 enhanced the invasive and migratory properties of MCF-7 cells or Az-521 cells (poorly metastatic gastric cancer cells). Interestingly, the PDZ2 domain of syntenin-1 was identified as being responsible for stimulating cell migration in this previous study^28^. This is consistent with our current data, which indicate that syntenin-1 interacts with syndecan-4 through the PDZ2 domain to block the functions of syndecan-4, thereby promoting migratory properties. Syntenin-1 expression is increased in highly metastatic melanoma cells^23^, and overexpression of tandem PDZ domains enhanced the migratory ability of cells more efficiently than overexpression of a single PDZ2 domain^29^. Collectively, the present and previous findings indicate that syntenin-1-mediated cell migration could be regulated by complicated and diverse mechanisms, in which the PDZ2 domain plays a major role.

The oligomeric organization of syntenin-1 is important for its interaction with the cytoplasmic domain of syndecan-4 and its ability to negatively regulate syndecan-4 signaling. A previous report proposed that full-length syntenin-1 undergoes a homotypic interaction, and that the tandem PDZ domains and at least a part of the NH_2_-terminal domain are essential for this interaction^30^. However, our data indicate that PDZ2 and the C-terminal region play critical roles in the functions of syntenin-1 (Fig. 6). In particular, the C-terminal region is essential for the assembly of syntenin-1 oligomers via interactions with syndecan-4 (Figs 5 and 6). Therefore, each PDZ domain alone might not be sufficient to confer the functions of syntenin-1 *in vivo*, and the overall integrity of syntenin-1 appears to be required. Our data provide strong evidence that the oligomerization of syntenin-1 is crucial for its ability to inhibit syndecan-4-mediated functions. In addition, QARF mutant syntenin-1 had a reduced ability to regulate cell migration and focal adhesion formation in REFs, compared to wild-type syntenin-1 (Fig. 5). Moreover, all of the syntenin-1 residues found herein to be involved in dimer formation and the interaction with syndecan-4 are completely conserved across vertebrates^9^. Therefore, the structure-function relationships reported herein may be widely applicable to other binding partners of the syntenin-1 PDZ2 domain. Since the dimerization of syntenin-1 affects its PDZ-mediated binding to syndecan-4, the syndecan-4 interaction might enable syntenin-1 to stably interact with a binding partner and effectively function as a negative regulatory scaffolding protein. Syntenin-1 dimerization is also necessary for the regulation of syndecan-4-mediated functions in fibroblasts. Since stable dimers of syndecan-4 on the cell membrane are probably the default state^31^, syntenin-1 dimers are well suited to suppress the functions of syndecan-4. Indeed, we found that dimerized syntenin-1 interacted with the cytoplasmic domain of syndecan-4 to rescue the tumor suppressor function of syndecan-4. Consistent with previous reports^23^, syntenin-1 expression is significantly increased in melanoma cells and contributes to enhancing the migratory potential of melanoma cells (Fig. 7). Syndecan-4, which acts as a tumor suppressor by regulating tumorigenic activities, is expressed in melanoma cells (Fig. 7). During the development of melanoma, increased syntenin-1 might contribute to the migration and invasion of melanoma cells. However, this effect will be more efficient if syntenin-1 overcomes the tumor suppression activity of other factors, such as syndecan-4. Here, we report for the first time that the syndecan-4/syntenin-1 interaction via syntenin-1 dimerization critically regulates melanoma cell migration and metastasis (Fig. 7). Therefore, future studies into the regulatory mechanisms governed by the syndecan-4/syntenin-1 complex may provide new insights into the metastatic potential of melanoma, possibly paving the way for the development of novel therapies aimed at reducing the metastasis of melanoma cells.

## Materials and Methods

### Antibodies

Monoclonal anti-HA, -His, -GST, and -β-actin antibodies and polyclonal anti-PKCα and -integrin β1 antibodies were purchased from Santa Cruz Biotechnology (Santa Cruz, CA, USA). Polyclonal anti-syndecan-4 were purchased from Abcam (Cambridge, UK), and monoclonal anti-paxillin was purchased from Upstate Biotechnology (Lake Placid, NY, USA).

### Cell Culture and Transfection

REF cells were maintained in alpha-modified Eagle medium (Gibco BRL) supplemented with 5% (v/v) fetal bovine serum (FBS), penicillin (10,000 units/ml), and streptomycin (1 mg/ml). HeLa and A375 cells were maintained in Dulbecco’s-modified Eagle medium supplemented with 10% FBS. Transient transfection was carried out using Effectene (Qiagen, Hilden, Germany) or Lipofectamine 2000 reagent (Invitrogen, Carlsbad, CA, USA) according to the manufacturer’s instructions.

### Peptide synthesis and purification

Peptides corresponding to the cytoplasmic domain and C2 region of syndecan-4, containing residues 175–202 (4L; RMKKKDEGSYDLGKKPIYKKAPTNEFYA) and 195–202 (4C2; APTNEFYA), respectively, of the rat protein were synthesized using an improved version of the solid-phase method using Fmoc chemistry (Anygen Inc., Kwangju, Korea).

### Crystallization and structure determination

Selenomethionine (Se-Met) labeled STNΔC was purified and concentrated to 15 mg/ml in 20 mM Tris-HCl (pH 7.5), 150 mM NaCl, 3 mM DTT, and 0.01% NaN_3_. Thereafter, it was mixed with syndecan peptide (4C2) at a 1:4 molar ratio and complex crystals were grown at 15 °C in a 1.5 μl micro batch under oil containing equal volumes of protein solution and mother liquor [0.2 M lithium sulfate, 0.1 M Bis-Tris (pH 6.5), and 25% (w/v) polyethylene glycol 3350]. Syntenin-1 was purified and concentrated to 15 mg/ml in 10 mM HEPES (pH 7.0), containing 150 mM NaCl, 5 mM DTT, and 0.01% NaN_3_ and syntenin-1 crystals were grown at 15 °C in a 1.5 μl micro batch under oil containing equal volumes of protein solution and mother liquor [0.1 M Bis-Tris (pH 6.5) and 25% (w/v) polyethylene glycol 3350]. In addition, purified syntenin-1 was mixed with 4C2 at a 1:4 molar ratio. Crystals of the syntenin-1/4C2 complex were grown in a 1.5 μl micro batch under oil containing equal volumes of protein solution and mother liquor [0.2 M lithium sulfate monohydrate, 0.1 M Tris hydrochloride (pH 8.5), and 30% (w/v) polyethylene glycol 4000]. All crystals were cryoprotected in reservoir solution supplemented with 20% (v/v) ethylene glycol. Diffraction data for the Se-Met STNΔC/4C2 complex, syntenin-1, and the syntenin-1/4C2 complex were collected at 2.5 Å, 1.9 Å, and 2.8 Å resolution, respectively, at beam line 5C at Pohang Accelerator Laboratory (PAL), Korea. Data were processed and scaled using HKL2000^32^. The space group of the Se-Met STNΔC/4C2 complex was fitted as P4_3_ (*a* = 64.2 Å, *b* = 64.2 Å, *c* = 201.77 Å, α = β = γ = 90°) and structure was determined by multi wave length anomalous dispersion in SOLVE^33^ and RESOLVE programs^34^. The space groups of both syntenin-1/4C2 complex and syntenin-1 were determined as P2_1_2_1_2_1_ (*a* = 67.53 Å, *b* = 88.53 Å, *c* = 90.37, *a* = 52.74 Å, *b* = 63.32 Å, *c* = 103.62 Å, α = β = γ = 90°) and structures were solved using the molecular replacement method with the program MOLREP. The crystallographic model was built using the COOT program^35^ and refined in REFMAC^36^. The final structures were analyzed using PROCHECK^37^. The statistics for structure refinement are summarized in Table 1. Values in parentheses are for highest-resolution shell and ramachandran plot was calculated by PROCHECK. The coordinates of syntenin-1, the syntenin-1/4C2 complex, and the STNΔC/4C2 complex have been deposited in the protein data bank (PDB) under pdb code 5G1E, 5G1D, and 5A2P, respectively.

### NMR experiments

All NMR experiments were performed in a mixture of 90% H_2_O and 10% ^2^H_2_O NMR buffer (50 mM NaPO_4_, 100 mM NaCl, 2 mM DTT and pH 7.0) at 298 K on a Bruker DRX 900 MHz equipped with a CryoProbe^TM^ system. The chemical shift in ^1^H was referenced directly to internal sodium 4,4-dimethyl-4-silapentane-1-sulfonate (DSS), and ^13^C and ^15^N were referenced indirectly to ^1^H. All spectra were processed using XWINNMR (Bruker Instruments, Karlsruhe, Germany) and NMRpipe/NMRDraw (Biosym/Molecular simulation, Inc. San Diego, CA, USA) software (Wuthrich, 1990; Delaglio *et al*. 1995).

### Immunoprecipitation

For immunoprecipitation, cells were lysed in 1% NP-40 lysis buffer (50 mM Tris, pH 8.0, 150 mM NaCl, 1% Nonidet P-40, 1 mM EDTA, 2 mM Na3VO4, and 10 mM NaF) containing protease inhibitor cocktail (1 μg/ml each of aprotinin, antipain, and pepstatin A; 20 μg/ml phenylmethylsulfonyl fluoride; and 5 μg/ml leupeptin). Each sample was incubated with polyclonal antibody against syndecan-4 for 2 hr at 4 °C, followed by 1 hr incubation with protein A-Sepharose beads (Sigma, St. Louis, MO, USA). Immune complexes were collected by centrifugation, washed three times with lysis buffer, resuspended in SDS sample buffer, and analyzed by SDS-PAGE.

### Cell fractionation

Hypoosmotic solution (20 mM Tris/HCl, pH 7.5, 2 mM 2-mercaptoethanol, 5 mM EGTA, and 2 mM EDTA) containing protease inhibitor cocktail was added to the culture plates. Cells were scraped off the plates and homogenized on ice. Homogenate was centrifuged at 13,000 × *g* for 15 min at 4 °C. The membrane fraction was collected by solubilizing the pellet in RIPA buffer (50 mM Tris, pH 8.0, 150 mM NaCl, 1 mM EDTA, 1% Nonidet P-40, 0.1% sodium dodecyl sulfate, 0.5% sodium deoxycholate, 10 mM NaF, and 2 mM Na_3_VO_4_) containing protease inhibitor cocktail, followed by centrifugation at 13,000 × *g* for 15 min at 4 °C. Equal amounts of lysates were resolved by SDS-PAGE, transferred to PVDF membranes, and probed with the indicated antibodies.

### *In vitro* PKC assay

For PepTag assay, PepTag C1 Peptide (Promega, Madison, WI, USA) was incubated in reaction buffer (20 mM HEPES, pH 7.4, 1 mM DTT, 10 mM MgCl_2_, and 1 mM ATP) in a final volume of 25 μl for 30 min at 30 °C. Reactions were stopped by heating at 95 °C for 10 min. Samples were separated on a 0.8% agarose gel at 100 V for 15 min. Phosphorylated peptide migrated toward the cathode (+) and nonphosphorylated peptide migrated toward the anode (−). The gel was photographed on a transilluminator.

### Immunofluorescence Microscopy

For immunostaining, REF cells were transfected with indicated cDNAs, fixed with 3.5% paraformaldehyde (PFA) in PBS at room temperature for 5 min, permeabilized with 0.1% Triton X-100 in PBS for 10 min, blocked with 0.5% bovine serum albumin in PBS for 1 hr, and incubated with paxillin antibody for focal adhesion identification in PBS for 24 hr. Slides were washed four times and incubated for 1 hr with 10 μg/ml secondary antibody, washed, and mounted with VECTASHIELD Mounting Medium (VECTOR Laboratories, Burlingame, CA). Slides were imaged by fluorescence microscopy using a CCD camera (Carl Zeiss, Göttingen, Germany).

### Migration assay

Gelatin (10 μg/ml) was added to each well of an 8-μm pore-size Transwell plate (Costar), and the membranes dried for 1 hr at room temperature. The Transwell plates were assembled in a 24-well plate and the lower chambers were filled with culture medium containing fibroblast growth factor (FGF)−2 (10 ng/ml). Cells were added to upper chambers and the plates were incubated at 37 °C in 5% CO_2_ for 24 hr. Cells that migrated to the lower surface of the filters were stained with 0.6% hematoxylin and 0.5% eosin and counted.

### Pull-down Assays

For biotinylated protein pull-down assays, biotinylated syndecan-4 cytoplasmic domain peptide (4 L) was prepared using a Mini-biotin-XX protein labeling kit (Invitrogen). Biotinylated 4 L (0.2 μM) was loaded on streptavidin magnetic beads (QIAGEN, Hilden, Germany), incubated with inversion at 4 °C and 0.5 μM each syntenin protein was added. Supernatant was removed after magnetic separation of the beads. Beads were washed twice with binding buffer. Bound proteins were solubilized in SDS sample buffer at 95 °C for 5 min, analyzed by SDS-PAGE on a 15% gel, and immunoblotted with specific antibodies. For His tag pull-down, recombinant His-tagged syntenin-1 proteins were purified on Ni-NTA agarose beads. Bound proteins were washed three times with lysis buffer and mixed with lysate of REF cells transfected with HA-tagged syntenin-1 or syntenin mutants. After incubation at 4 °C on a rotator for 2 hr, the precipitated complex was eluted with SDS-PAGE sample buffer, resolved by SDS-PAGE, and immunoblotted with anti-HA antibody.

### Size exclusion gel chromatography

The protein domains nPDZ1, PDZ1, PDZ2, PDZ2C, STNΔC, and syntenin-1 were purified by Ni-NTA affinity chromatography. Molecular masses and folding state of each construct were analyzed by size exclusion gel chromatography with HiLoad Superdex 75 columns (Pharmacia) in buffer containing 20 mM sodium phosphate, 100 mM NaCl, 0.01% NaN_3_, and 5 mM dithiothreitol, at pH 7.0.

### Lung metastasis model

C57BL/6 mice were maintained under conventional housing conditions using a chamber system. Male mice aged 7 weeks (*n* = 4~6 animals per group) were injected with 1 × 10^5^ B16F10 cells stably expressing syndecan-4, syntenin, or syndecan-4/syntenin-1 (coexpression of SDC4 and STN) in 0.15 ml PBS containing 0.05% BSA via tail vein. 13 days later, the metastatic lesions on the lung were photographed and counted the numbers of metastatic nodules on their surface. All animal experiments were approved by the Animal Research Committees of Kyungpook National University and were performed in accordance with the Guide for the Care and Used of Laboratory Animals of Kyungpook National University.

### Statistical Analysis

Data are presented as the means of at least three independent experiments. Statistical analysis was performed using an unpaired Student’s *t* test. A *p* value < 0.01 was considered statistically significant.

## Additional Information

**How to cite this article**: Choi, Y. *et al*. New structural insight of C-terminal region of Syntenin-1, enhancing the molecular dimerization and inhibitory function related on Syndecan-4 signaling. *Sci. Rep.* **6**, 36818; doi: 10.1038/srep36818 (2016).

**Publisher’s note:** Springer Nature remains neutral with regard to jurisdictional claims in published maps and institutional affiliations.

## Supplementary Material

Supplementary Information

## Figures and Tables

**Table 1:** Data collection and refinement statistics.

	**Syntenin-1/4C2**			**Syntenin-1**			**STNΔC/4C2**		
**Data collection**									
Space group	*P2*_*1*_*2*_*1*_*2*_*1*_			*P2*_*1*_*2*_*1*_*2*_*1*_			P4_3_		
Cell dimensions									
* a, b, c* (Å)	67.525	88.529	90.368	52.740	63.318	103.620	64.2	64.2	201.767
* α, β, γ* (°)	90.00	90.00	90.00	90.00	90.00	90.00	90.00	90.00	90.00
Resolution (Å)	50–2.8			50–1.9			20.0–2.5		
I / σI^*^	24.18 (4.3)			41.18 (4.3)			13.96 (2.57)		
*R*_merge_ (%)	7.4 (28.8)			5.2 (34.8)			8.8 (38.1)		
Completeness (%)	97.6 (93.9)			97.9 (99.3)			100 (100)		
Redundancy	8.8 (4.8)			10.7 (6.7)			5.5		
**Refinement**									
Resolution (Å)	30–2.8			30–1.92			19.44–2.5		
No. reflections	12615			27216			26732		
*R*_work_ / *R*_free_	19.42/23.49			23.15/27.9			23.4/28.6		
No. atoms									
* *Protein	2679			2560			5097		
* *Water				147			105		
*B*-factors	37.64			30.099			36.8		
R.m.s. deviations									
* *Bond lengths (Å)	0.030			0.017			0.017		
* *Bond angles (°)	1.989			1.767			1.500		
Ramachandran plot (%)									
* *Most favored regions	93			88.6			93.6		
* *Allowed regions	7			10.3			6.4		
* *Disallowed regions	0			1			0		

**Table 2:** Analyzed molecular weight through the size exclusion gel chromatograph.

	**M.W (KDa)**	**Elution volume (ml)**	**Calculated M.W (KDa)**	**State**
**STN**	21.8	63	38.8	dimer
**QARF**	20.8	72	22.3	monomer

**Table 3:** Analyzed molecular weight through the size exclusion gel chromatograph.

	**M.W (KDa)**	**Elution volume (ml)**	**Calculated M.W (KDa)**	**State**
**STN**	21.8	61	45.14	dimer
**ΔC**	17.5	78	15.84	monomer
**nPDZ1**	9.4	83	11.67	monomer
**PDZ1**	8.8	86	9.7	monomer
**PDZ2**	8.7	87	9.12	monomer
**PDZ2c**	11.7	73	21.58	dimer
