# Supplementary material for: New structural insight of C-terminal region of Syntenin-1, enhancing the molecular dimerization and inhibitory function related on Syndecan-4 signaling
Source: Sci Rep. 2016 Nov 10;6:36818. doi: 10.1038/srep36818 (PMC5103296; doi:10.1038/srep36818)
Supplement: Supplementary Information [file srep36818-s1.pdf]

# New structural insight of C-terminal region of Syntenin-1, enhancing the molecular dimerization and inhibitory function related on Syndecan-4 signaling

Youngsil Choi[1,†], Ji-Hye Yun[2,†], Jiho Yoo[3], Inhwan Lee[2], Heeyoun Kim[2], Hye-Nam Son[4], In-San Kim[4], Ho Sup Yoon[5], Pascale Zimmermann[6], John R. Couchman[7], Hyun-Soo Cho[3], Eok-Soo Oh[1*], and Weontae Lee[2*]

[1]Department of Life Sciences, Division of Life and Pharmaceutical Sciences and the Research Center for Cellular Homeostasis, Ewha Womans University, Seoul 120-750 Korea, [2]Department of Biochemistry, College of Life Science & Biotechnology, Yonsei University, Seoul 120-749 Korea, [3]Department of Biology, College of Life Science & Biotechnology, Yonsei University, [4]Biomedical Research Institute, Korea Institute of Science and Technology, Seoul 136-791, Republic of Korea, [5]Division of Structural and Computational Biology, School of Biological Sciences, Nanyang Technological University, Singapore, [6]Laboratory for Glycobiology, University of Leuven & Flanders Interuniversity Institute for Biotechnology, Leuven, Belgium, [7]Department of Biomedical Sciences, University of Copenhagen, Biocenter, 2200 Copenhagen, Denmark,

*To whom correspondence should be addressed:

Weontae Lee, Department of Biochemistry, College of Life Science & Biotechnology, Yonsei University E-mail: wlee@spin.yonsei.ac.kr; Eok-Soo Oh, Dept. of Life Sciences, Ewha Womans University, Daehyun-dong, Seodaemoon-Gu, Seoul 120-750, Korea, Phone: +82-2-3277-3761; Fax: +82-2-3277-3760; E-mail: OhES@ewha.ac.kr.

†Co-first Authors

Running title: Syntenin-1, the inhibitory scaffolding protein

## Supplementary information

### Supplementary material and methods

*Materials and Antibodies*

Polyclonal anti-vinculin was purchased from Santa Cruz Biotechnology (Santa Cruz, CA, USA). Monoclonal anti-phospho-PKCα (E-195) was purchased from Abcam (Cambridge, UK). [$\gamma$-$^{32}$P] ATP was purchased from Pharmacia Biotech (Arlington Heights, IL, USA). Histone III-S and other chemicals were purchased from Sigma (St. Louis, MO, USA).

*Immunoblotting*

For immunoblotting, cells were lysed in RIPA buffer (50 mM Tris, pH 8.0, 150 mM NaCl, 1 mM EDTA, 1% Nonidet P-40, 0.1% sodium dodecyl sulfate (SDS), 0.5% sodium deoxycholate, 10 mM NaF, and 2 mM $Na_3VO_4$) containing protease inhibitor cocktail (1 μg/ml each of aprotinin, antipain, and pepstatin A; 20 μg/ml phenylmethylsulfonyl fluoride; and 5 μg/ml leupeptin). Lysates were clarified by centrifugation at 13,000 × *g* for 15 min at 4°C, denatured in SDS sample buffer, boiled, and analyzed by SDS-polyacrylamide gel electrophoresis.

*FRET Assays*

For FRET experiments, DNA encoding syndecan-4 and the PKCα catalytic domain (PKM) were ligated into plasmids pEYFP-N1 and pECFP-C1 (Clontech, Mountain View, CA, USA), respectively. Syntenin-1 in a pET-21b plasmid was subcloned into plasmid pXJ-Flag-S. All expression plasmids were verified by DNA sequencing. HeLa cells were seeded in a 6-well tissue culture plate with a coated glass coverslip at a density of approximately $2 \times 10^5$ cells/well. Cells were transfected with expression plasmids using Lipofectamine 2000 reagent (Invitrogen) according to the manufacturer's instructions. Cells were fixed with 3.7% PFA in PBS for 10 min and mounted on slides using Slowfade antifade kit (Molecular Probes, Eugene, OR, USA). A Zeiss LSM510 META confocal microscope (Zeiss, Oberkochen, Germany) was employed for FRET analysis with acceptor photobleaching. Briefly, images with the following configuration were recorded: CFP: excitation wavelength, 458 nm/emission filter, BP 475-525 nm; YFP: excitation wavelength, 514 nm/emission filter, LP560. Cells were visualized with an oil immersion 63× objective. Cells were bleached in the YFP channel by scanning a region of

interest (ROI) 10 times using the 514 argon laser line at 75% intensity. The CFP signal of the ROI was acquired before and after photobleaching. FRET efficiency ($E_F$) was calculated as a percentage using the equation $E_F = (I_6 – I_5) \times 100/I_6$, where $I_n$ is the CFP intensity at the $n^{th}$ time point. Bleaching was performed between the $5^{th}$ and $6^{th}$ time points. Unbleached regions were calculated as background control to determine CFP fluctuations during FRET analysis: $C_F = (I_6 – I_5) \times 100/I_6$.

*Protein expression and purification*

Syntenin-1 DNA was obtained from Mingjie Zhang of Hong Kong University of Science and Technology. DNA fragments encoding the nPDZ1 (residues 108-193), PDZ1 (residues 113-193), PDZ2 (residues 194-274), PDZ2C (residues 194-300) and STNΔC (residues 108-274) domains of the syntenin-1 (108-300) were inserted into modified pET-21b with a $(His)_6$-tag at the N-terminus (Pharmacia Biotech). The vectors were transformed into *Escherichia coli* BL21 (DE3) cells (Invitrogen) for fusion protein expression. 1 mM isopropyl β-D-thiogalactopyranoside (IPTG) was added to the growth medium to induce protein expression after cell density reached $OD_{600}$ of 0.6. The $(His)_6$-tag of each construct was cleaved by bovine thrombin (Pharmacia, Uppsala, Sweden) digestion for 15 hr in batches. Further purification was carried out by gel filtration chromatography on HiLoad Superdex 75 (Pharmacia) in buffer containing 20 mM sodium phosphate, 100 mM NaCl, 0.01% $NaN_3$, and 5 mM dithiothreitol at pH 7.0 for PDZ1, PDZ2, PDZ2C and STNΔC, and at pH 6.5 for syntenin-1. Purified protein was concentrated with Amicon (Millipore, Bedford, MA, USA) 5 kD and 10 kD cut-off filters. Freshly prepared recombinant proteins were used in all experiments. For NMR spectroscopy, protein samples uniformly labeled with [$^2H/^{15}N$] and [$70\%^2H/^{13}C/^{15}N$] were prepared by growing cells in $D_2O$ M9 minimal medium containing $^{15}NH_4Cl$, with or without $^{13}C_6$-D-glucose, as the sole sources of nitrogen and carbon. Uniformly [$^{15}N$]- and [$^{13}C/^{15}N$]-labeled proteins were also prepared by growing cells in M9 media containing $^{15}NH_4Cl$, with or without $^{13}C_6$-D-glucose, as the sole sources of nitrogen and carbon. NMR samples were of approximately 0.5-1.5 mM concentration in 90% $H_2O$/10% $^2H_2O$ and were loaded into 5-mm symmetrical micro cells (Shigemi$^{TM}$).

*Surface plasmon resonance analysis*

SPR measurements were performed with the BIACORE BIOSENSOR and BIAEVALUTION V3.1 interactive software (Pharmacia Biosensor, Uppsala, Sweden). His-

4L was immobilized at a flow rate of 10 μl/min using PBS (pH 6.5) containing 2 mM DTT. Equal volumes of 0.1 M *N*-hydroxysuccinimide and 0.4 M *N*-ethyl-*N'*-(3-diethlyaminopropyl) carbodiimide were mixed and injected into a CM5 sensor chip to activate the carboxymethylated dextran surface. The volume was adjusted to achieve immobilization levels of His-4L, making 6000 resonance units. After injection of His-4L, the residual NHS esters were deactivated with 25 μl ethanolamine (1 M, pH 8.5). STN was injected into the immobilized His-4L sensor chip at a concentration of 5.2-166.8 μM and flow rate of 30 μl/min.

*Lymph node metastasis model*

C57BL/6 mice were maintained under conventional housing conditions using a chamber system. Male mice aged 7 weeks ($n$ = 4~6 animals per group) were injected in the right hind footpad with $3 \times 10^5$ B16F10 cells stably expressing syndecan-4 and mock vector (control) in 0.05 ml PBS containing 0.05% BSA. Two weeks later, the metastatic popliteal lymph nodes were removed and photographed, and the sizes were measured by area analysis using the ImageJ software. All animal experiments were approved by the Animal Research Committees of Kyungpook National University and were performed in accordance with the Guide for the Care and Used of Laboratory Animals of Kyungpook National University.

**Supplementary Figure legends**

**Figure S1: The Superposition of syntenin-1 and syntenin-1/4C2 complex** (A) X-ray structure of free syntenin-1 is superimposed with that of the syntenin-1/4C2 complex. Syndecan-4-bound syntenin-1 and free syntenin-1 are shown in purple and blue, respectively. (B and C) Upon syndecan-4 peptide binding to syntenin-1, helix 2 of PDZ1 domain, helix 4 of PDZ2 domain are shifted by 4° in opposite directions

**Figure S2: Syntenin-1 as negative regulator of syndecan-4.** (A) REF cells were co-transfected with syndecan-4 (SDC4) and either vector or HA-tagged syntenin-1 (HA-STN). REF cell extracts were immunoprecipitated with anti-syndecan-4 antibody, and immune complexes were blotted with either anti-PKCα or anti-HA antibody. (B) HeLa cells were co-transfected with syndecan-4-YFP and PKM-CFP in the presence or absence of FLAG-tagged-STN. To visually monitor the interaction of SDC4 with PKM, FRET measurements were performed. Representative images of donor (CFP) and acceptor (YFP) before and after acceptor photobleaching are shown. (C) FRET efficiency was measured as CFP fluorescence intensity in bleached region (Ef) and unbleached region as control (Cf). (D) REF cells were co-transfected with SDC4 and either vector or HA-tagged STN. Cells were lysed and protein phosphorylation was determined by blotting with anti-phospho-PKCα antibody. α-PKCα served as loading control. (E) REF cells were co-transfected with SDC4 and either vector or STN. After 24 hr, cells were fixed with 3.5 % PFA for 5 min, extracted with 0.1% Triton X-100 in PBS for 10 min, and stained with anti-paxillin antibodies (*top panel*). Percentages of cells showing focal adhesions (mean±SE; vector transfectants set as 100%, *bottom panel*). *, $p<0.01$ vs. syndecan-4. (F) Transwell migration assays were performed using FGF-2 as a chemoattractant in the lower chamber. Transfected cells were allowed to migrate on gelatin-coated Transwell plates for 24 hr. Results are representative of three independent experiments.

**Figure S3 : Molecular interaction of syntenin-1 and syndecan-4 cytoplasmic domain mediating PDZ2 domain** (A) Surface plasmon resonance sensorgrams showing the interaction between syndecan cytoplasmic domain and syntenin-1. Profiles of syntenin-1 binding to syndecan cytoplasmic domain (4L) immobilized on carboxymethyl dextran surface. The superimposed curves are derived from experiments with syntenin-1 concentrations of 1.3, 2.6, 5.2, 10.4, 20.8, 41.7, 83.4, 166.8 and 333.6 μM (from bottom to top), respectively. The

observed signals are presented as a plot of resonance units (RUs) vs. time. (B and C) NMR mapping of syntenin-1/4L interactions in solution. (B) $^{15}$N-labeled PDZ1 domain of syntenin-1 was titrated with 4L. Bar diagram shows chemical shift changes of PDZ1 domain residues upon 4L binding. (C) $^{15}$N-labeled PDZ2 domain of syntenin-1 was titrated with 4L. Bar diagram shows chemical shift changes of PDZ2 domain upon syn-4L binding. Residues interacting with 4L, determined based on the chemical shift titration, are shown in red. The perturbed regions upon 4L binding are displayed in red color on a ribbon plot.

**Figure S4 : The inhibitory role of PDZ2 domain-mediated syntenin-1 on syndecan-4 functions** (A) REF cells co-transfected with syndecan-4 (SDC4) and vector, HA-tagged STN, -PDZ1, or -PDZ2 were immunoprecipitated with anti-syndecan-4 antibody. Immune complexes were blotted with anti-PKCα. (B) Cells were co-transfected with the indicated cDNAs and the amount of PKCα in the membrane fraction was determined by immunoblotting with anti-PKCα antibody. Integrin β1 served as a loading control. (C) PKC assays were performed as described in Experimental Procedures with purified STN or deletion mutants (PDZ1 or PDZ2). Relative activity is presented as mean±SE (n=5) compared with that in the absence of syndecan-4 proteins. *, p<0.01; **, p<0.05. (D) REF cells were co-transfected with wild-type SDC4 and HA-tagged STN or deletion mutants (PDZ1 or PDZ2). Total RNA was extracted from cells transfected with the indicated cDNAs and mRNA was analyzed by RT-PCR *(top panel)*. β-actin mRNA served as loading control. Cell lysates were separated by electrophoresis on 15% SDS-PAGE gels and syntenin-1 expression was analyzed using anti-HA antibody *(middle panel)*. Migration assays were performed as described in Figure 3e, using Transwells coated with gelatin (10 µg/ml) *(bottom panel)*. Shown is the relative amount of cell migration. Results from three independent experiments are combined. *, p<0.01; **, p<0.05 vs. syndecan-4.

**Figure S5 : The effects of syndecan-4 on popliteal lymph node metastasis *in vivo.*** (A and B) A375 melanoma cells were transfected with the indicated cDNAs and syndecan-4 mRNA expression was evaluated by RT-PCR and western blotting (top). Transfected cells were allowed to migrate on gelatin-coated (10 µg/ml) Transwell plates, and migrated cells were stained with hematoxylin and eosin, and counted (bottom). **, p<0.01 vs. Vec (C) C57BL/6 mice were i.v. injected with B16F10 cells (1×$10^5$ cells) stably expressing the indicated cDNAs. 14 days after injection of B16F10 cells, surface metastatic nodules in lung were photographed

(top) and counted (bottom). *, p<0.01 vs. Vec (D) C57BL/6 mice were injected into right footpad with B16F10 cells ($3 \times 10^5$ cells) stably expressing syndecan-4. 2 weeks later, popliteal lymph node were removed and photographed (top) and calculated the area of each lymph node using ImageJ software (bottom). Scale bar represents 1 mm. *, p<0.01 vs. Vec.

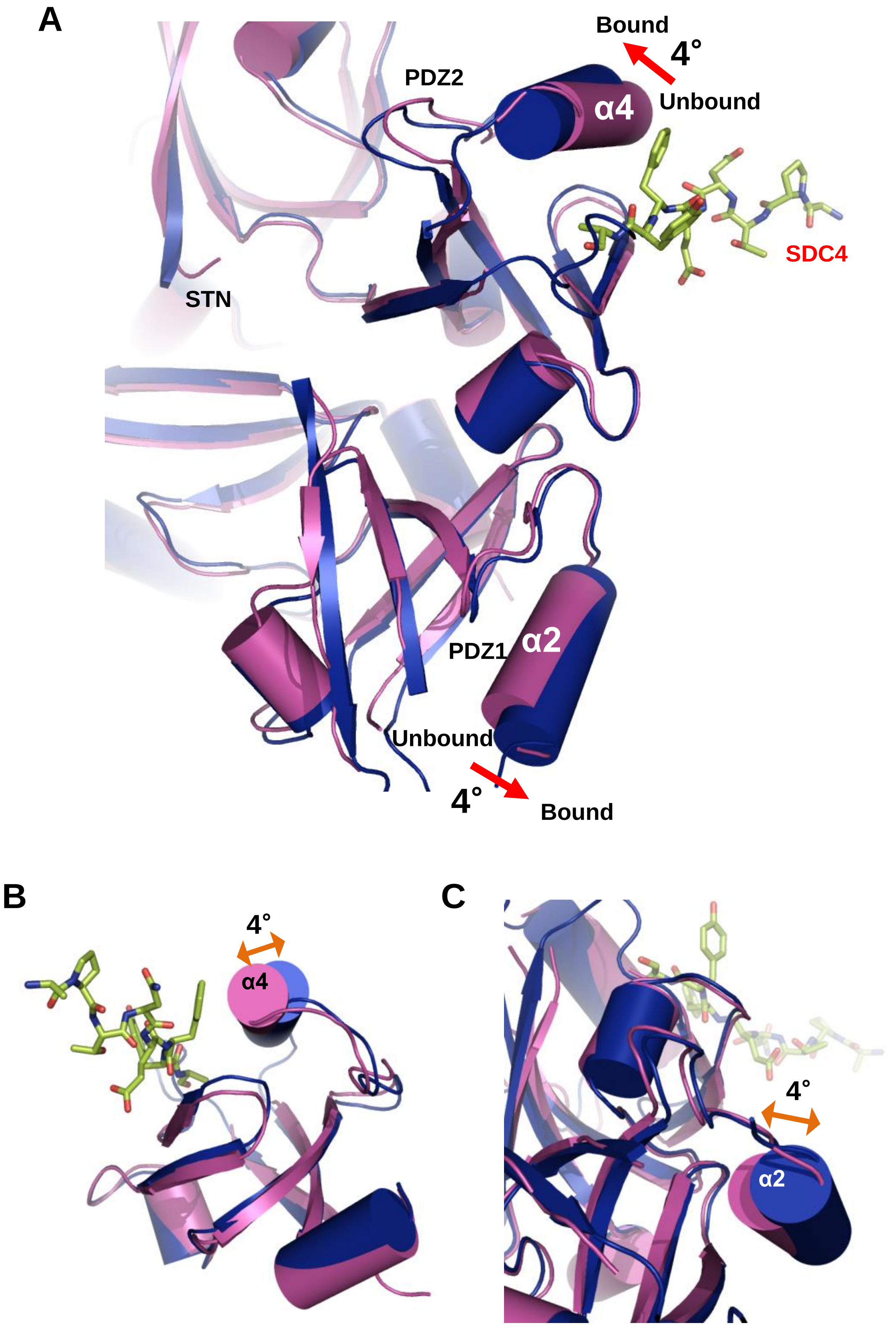


Supplementary Figure S1

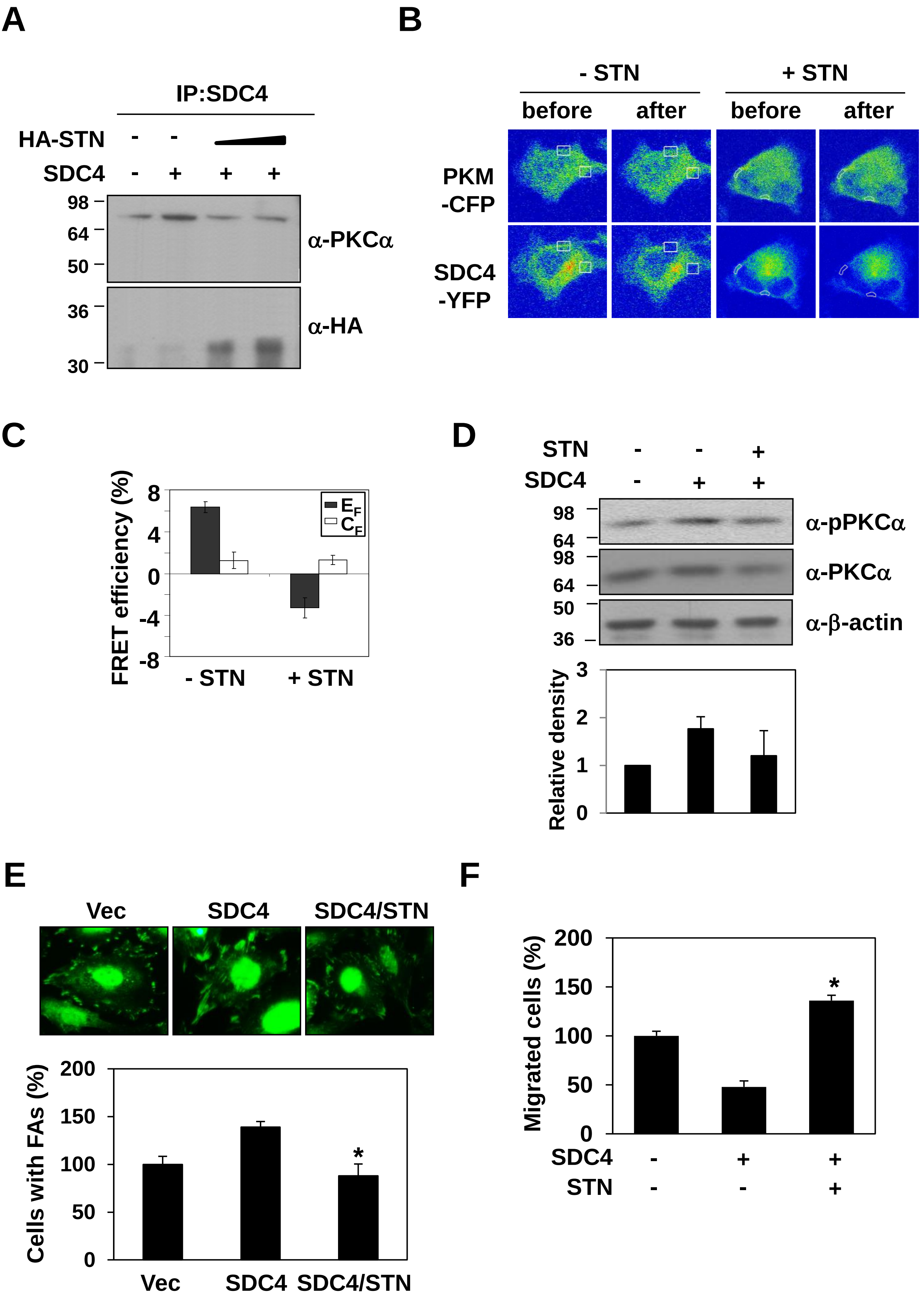


# Supplementary Figure S2

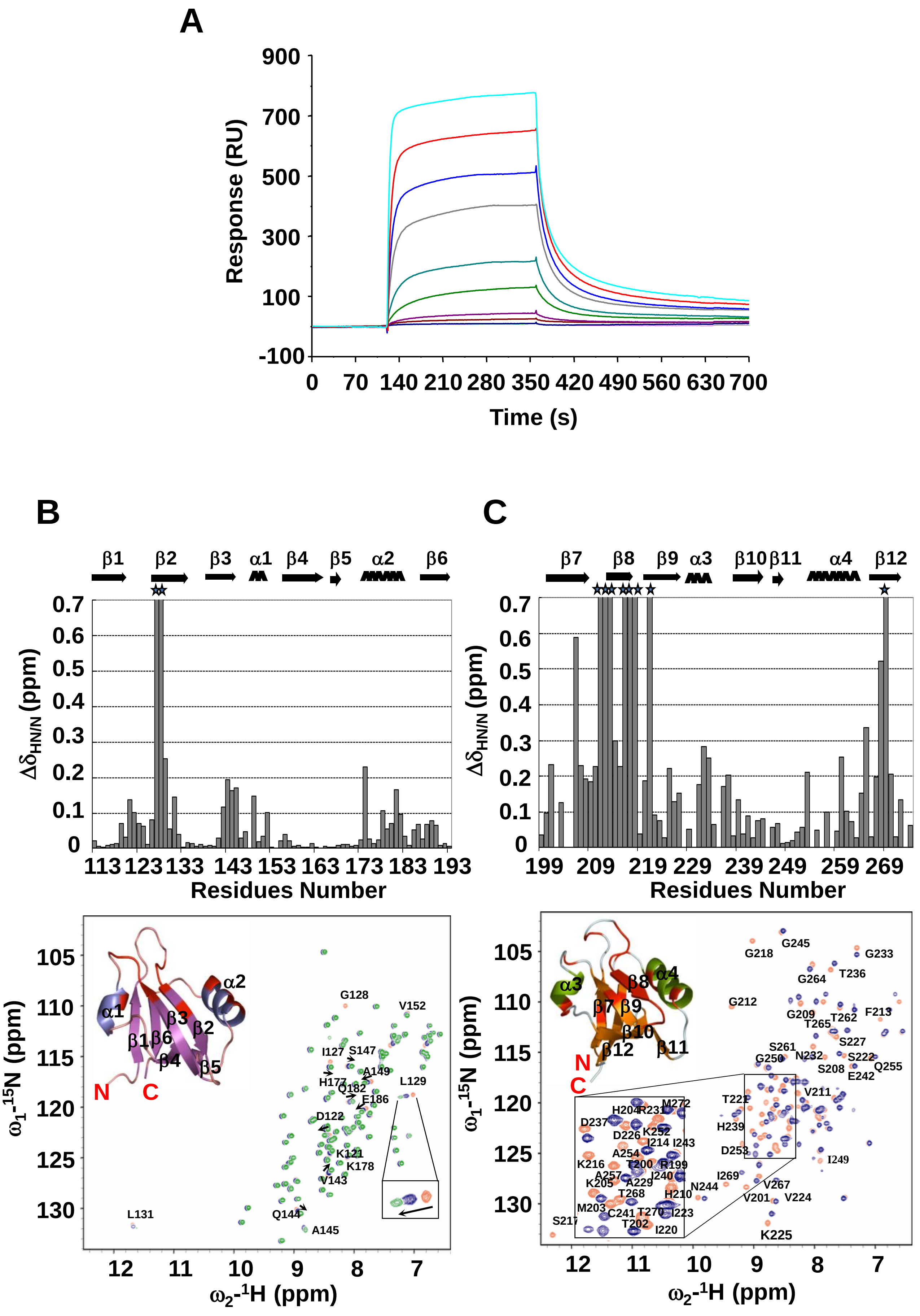


Supplementary Figure S3

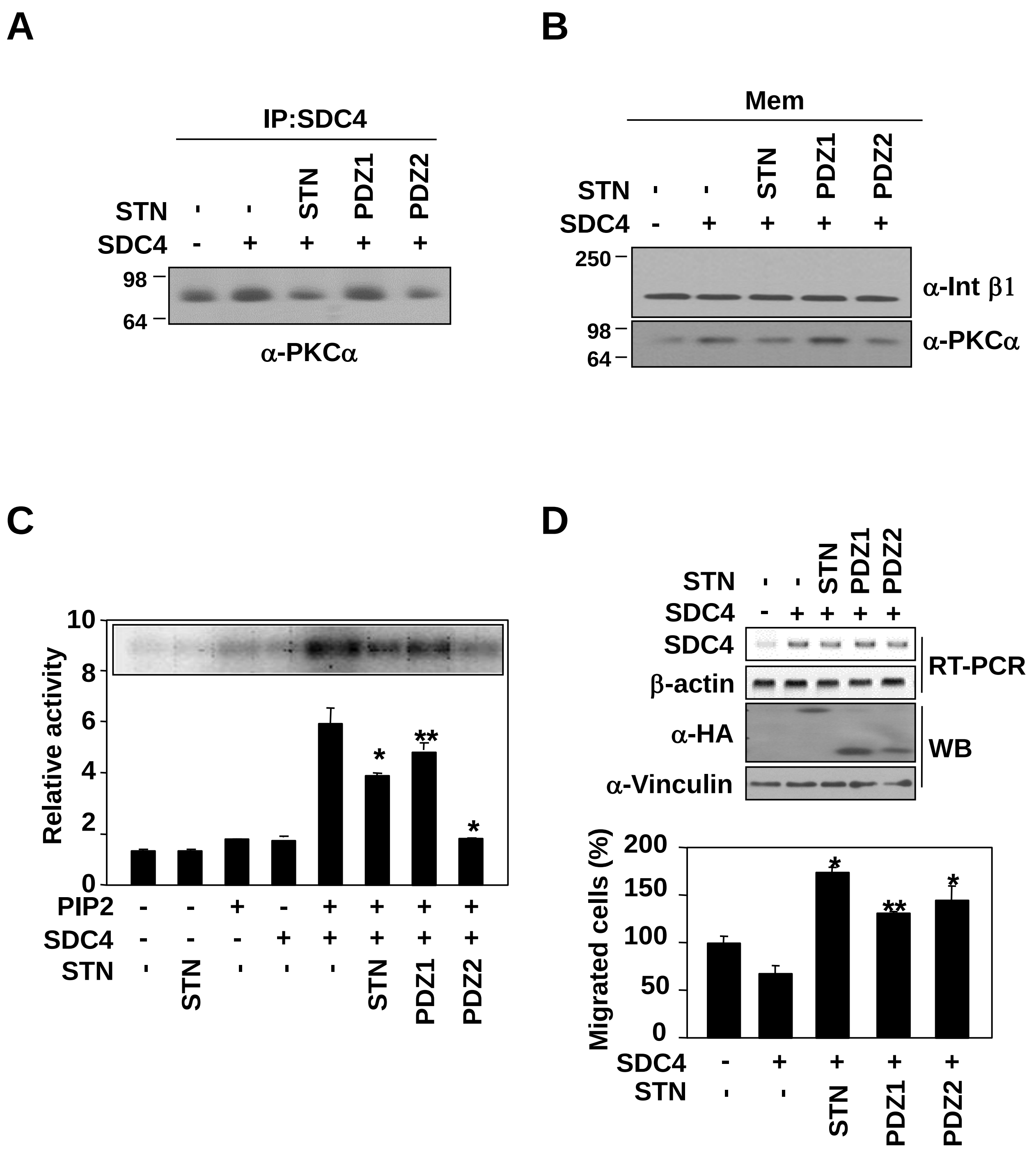


Supplementary Figure S4

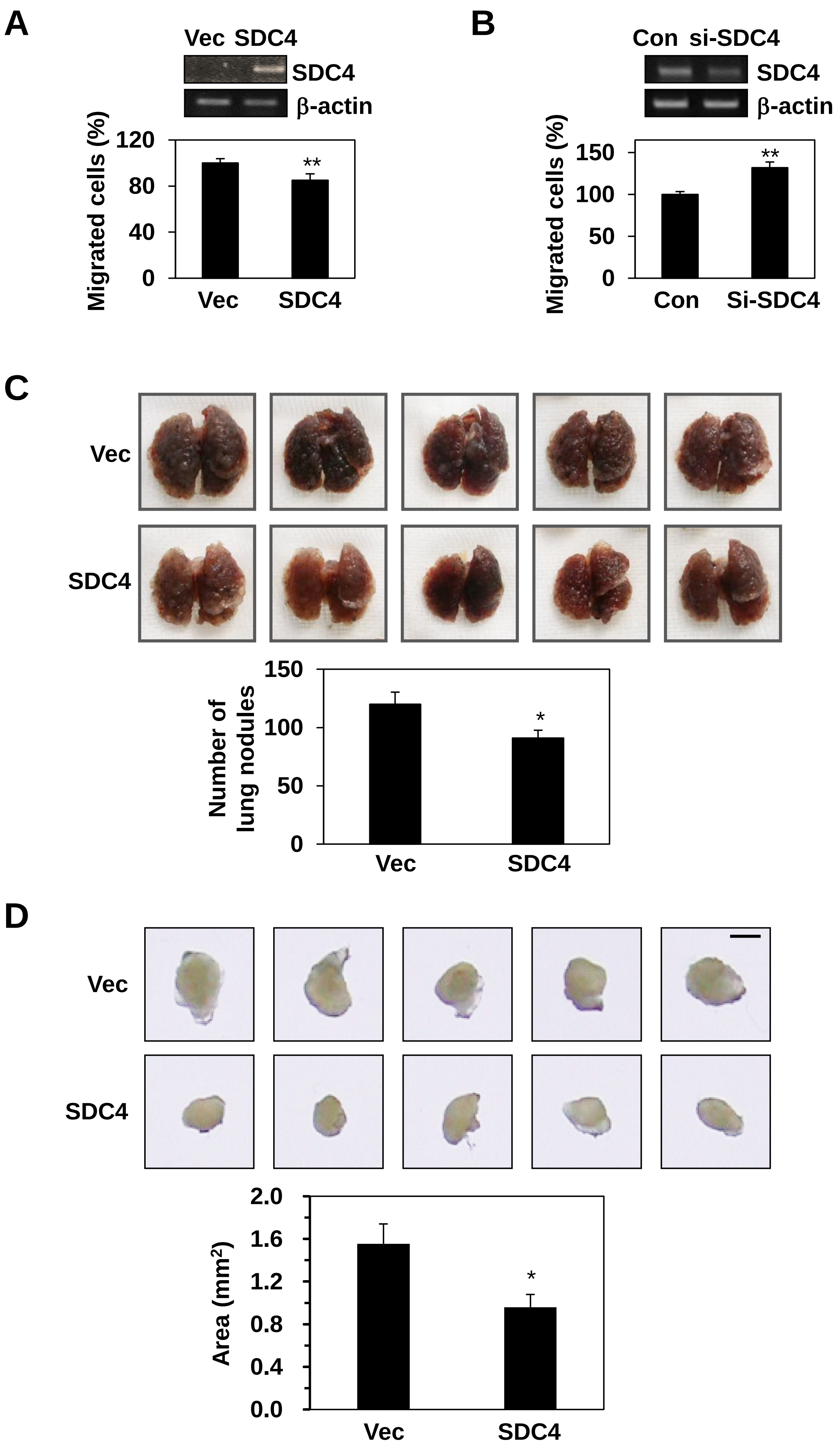


**Supplementary Figure S5**
